# Supplementary material for: Prdm16 regulates the postnatal fate of embryonic radial glia via Vcam1-dependent mechanisms
Source: Nat Commun. 2025 Jul 19;16:6659. doi: 10.1038/s41467-025-60895-y (PMC12276310; doi:10.1038/s41467-025-60895-y)
Supplement: Supplementary file 1 — Supplementary Information [file 41467_2025_60895_MOESM1_ESM.pdf]

Prdm16 regulates the postnatal fate of embryonic radial glia via Vcam1-dependent mechanisms

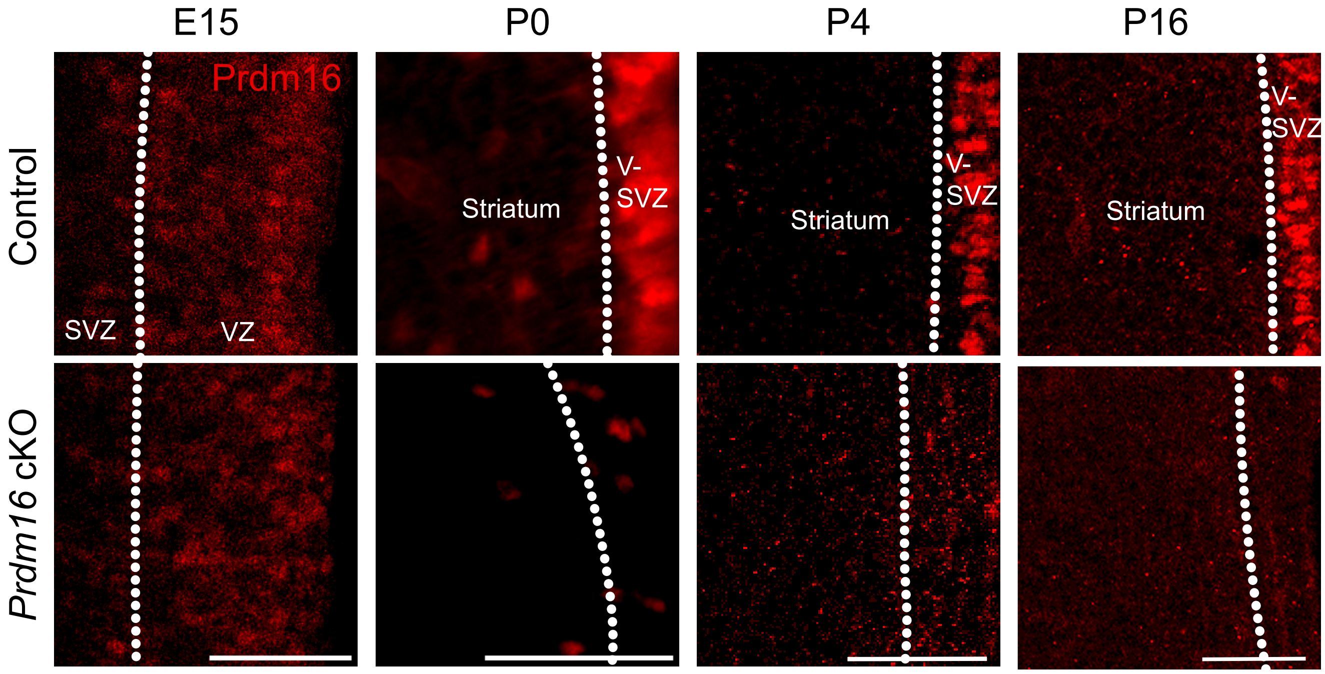

Supplementary Figure 1. Prdm16 immunofluorescence in the V-SVZ and striatum of *Prdm16* cKO and control mice from E15 to P16. The dashed lines delineate the boundaries between the SVZ and VZ (E15) or between the V-SVZ and striatum (P0-P16). All Scale bars are 50 μm. All experiments were repeated more than three times.

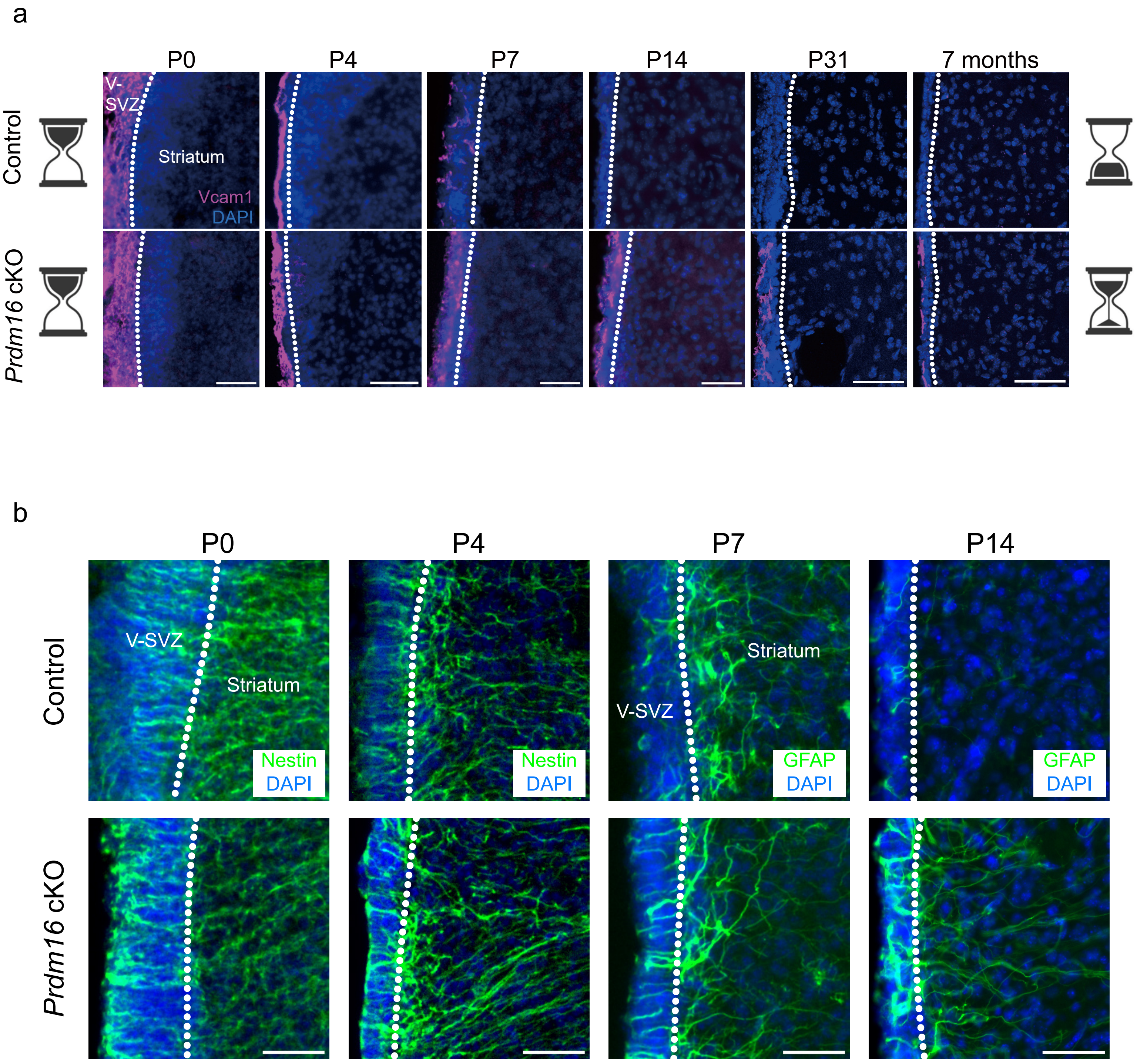

Supplementary Figure 2. Phenotype onset in *Prdm16* cKO mice.
a Vcam1 immunofluorescence decreases with age in the V-SVZ in control mice but remains high in *Prdm16* cKO mice. Immunofluorescence of Vcam1 in the V-SVZ at a series of postnatal ages. Dotted lines delineate the boundaries between V-SVZ and striatum. Scale bars: 50 µm.
b Radial glial processes marked by Nestin and GFAP immunofluorescence decrease postnatally and mostly disappear by P15 in control mice. In contrast, radial glial processes persist in *Prdm16* cKO mice. The difference becomes detectable by P14. Scale bars: 50 µm.
All experiments were repeated more than three times.

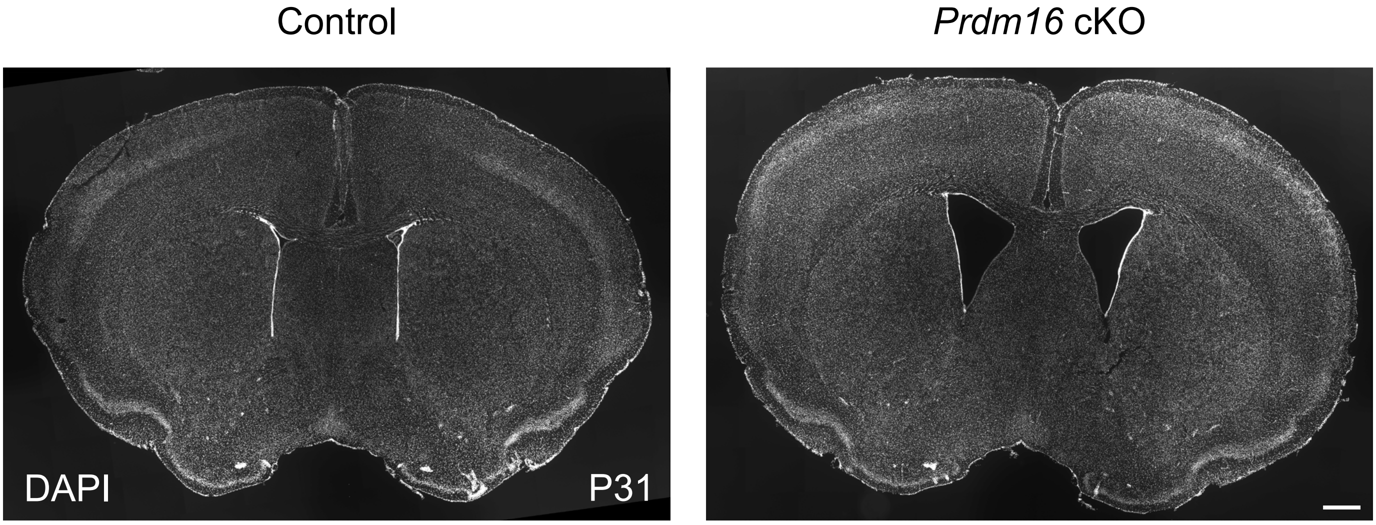

Supplementary Figure 3. ***Prdm16*** **cKO mice exhibit hydrocephaly.**
Scale bar: 500 µm. Brain sections from P31 mice are shown. The experiment was repeated more than three times.

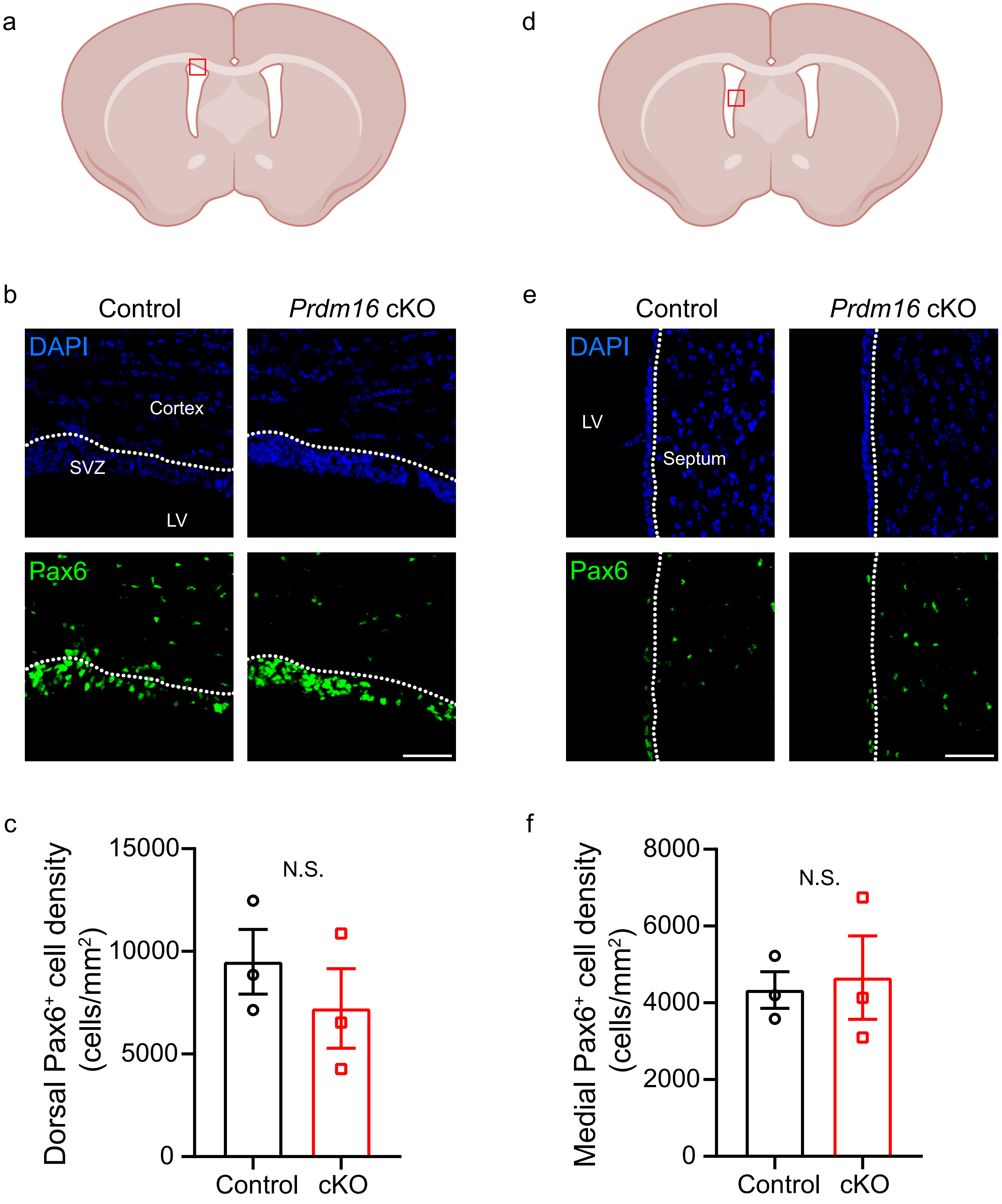

Supplementary Figure 4. No change in the density of Pax6$^+$ NSCs in the dorsal and medial walls of the lateral ventricle in *Prdm16* cKO mice. a, d Regions imaged and quantified are indicated with red squares. **b, e** Pax6 immunofluorescence in the dorsal (b) and medial (e) sides of the lateral ventricle at P30. Dotted lines delineate the boundaries between V-SVZ and lateral ventricle (LV). Scale bars: 50 µm. **c, f** Quantification of Pax6$^+$ cell density. Dorsal: n=3 mice per genotype, p=0.4162, two tailed Welch's t-test. Medial: n=3 mice per genotype, p=0.8051, two tailed Welch's t-test.

a and **d** were Created in BioRender. Zhang, Y. (2025) https://BioRender.com/y53n311

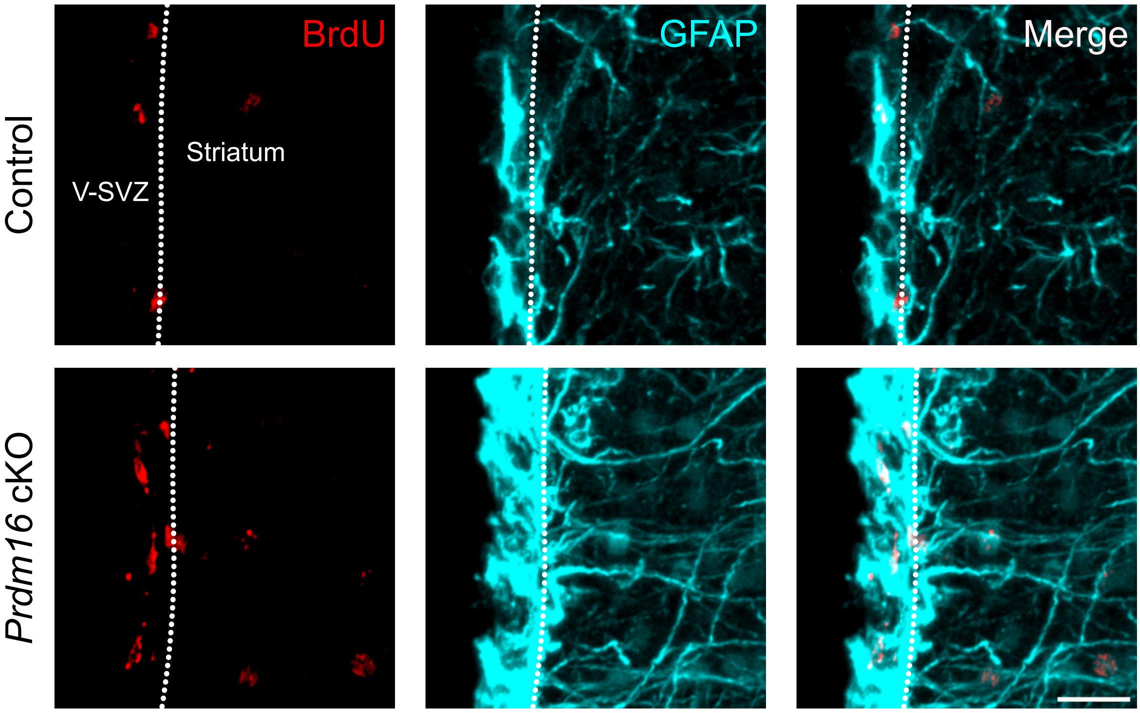

Supplementary Figure 5. Colocalization of BrdU with NSC marker GFAP
BrdU was injected daily between E15.5 and E17.5. Mice were sacrificed at P21. Red: BrdU. Light blue: GFAP. Scale bar: 20 μm. The experiment was repeated more than three times.

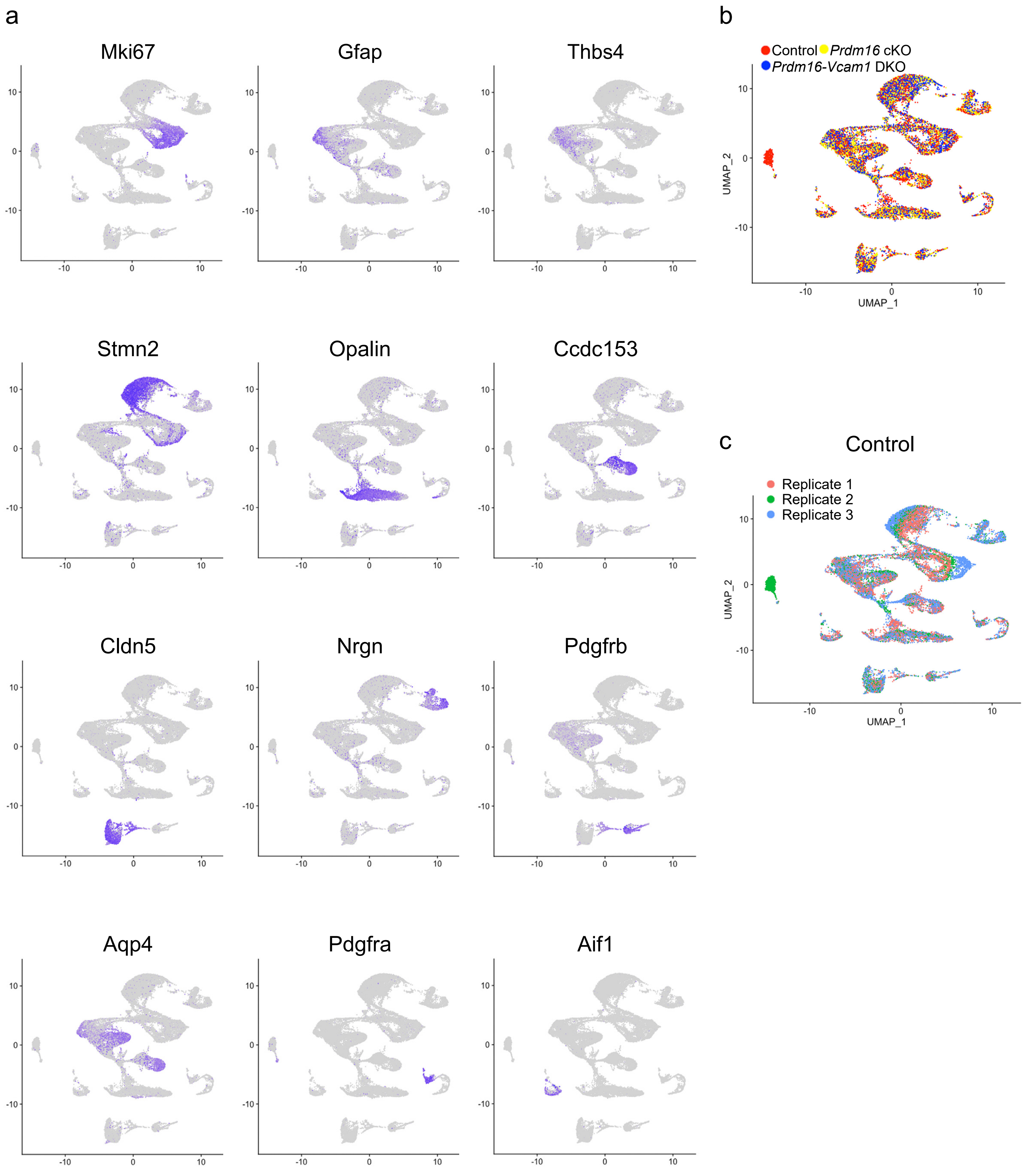

Supplementary Figure 6. Expression of cell-type markers by major cell clusters in the scRNA-seq dataset

a We used the expression of the following cell-type markers to identify cell clusters in our one-month-old V-SVZ scRNA-seq dataset: transient amplifying precursors (TAP, *Mki67*), neural stem cells (*Gfap*, *Thbs4*), neuroblasts (*Stmn2*), oligodendrocytes (*Opalin*), ependymal cells (*Ccdc153*), epithelial cells (*Cldn5*), neurons (*Nrgn*), pericytes (*Pdgfrb*), astrocytes (*Aqp4*), oligodendrocyte precursor cells (*Pdgfra*), microglia (*Aif1*). The expression of the marker genes on UMAP is shown.

b, c Control, *Prdm16* cKO, and *Prdm16-Vcam1* DKO samples contain cells from all clusters except for cluster 6 (red in b), which is only detected in one of the three control samples. We excluded cluster 6 from further analyses.

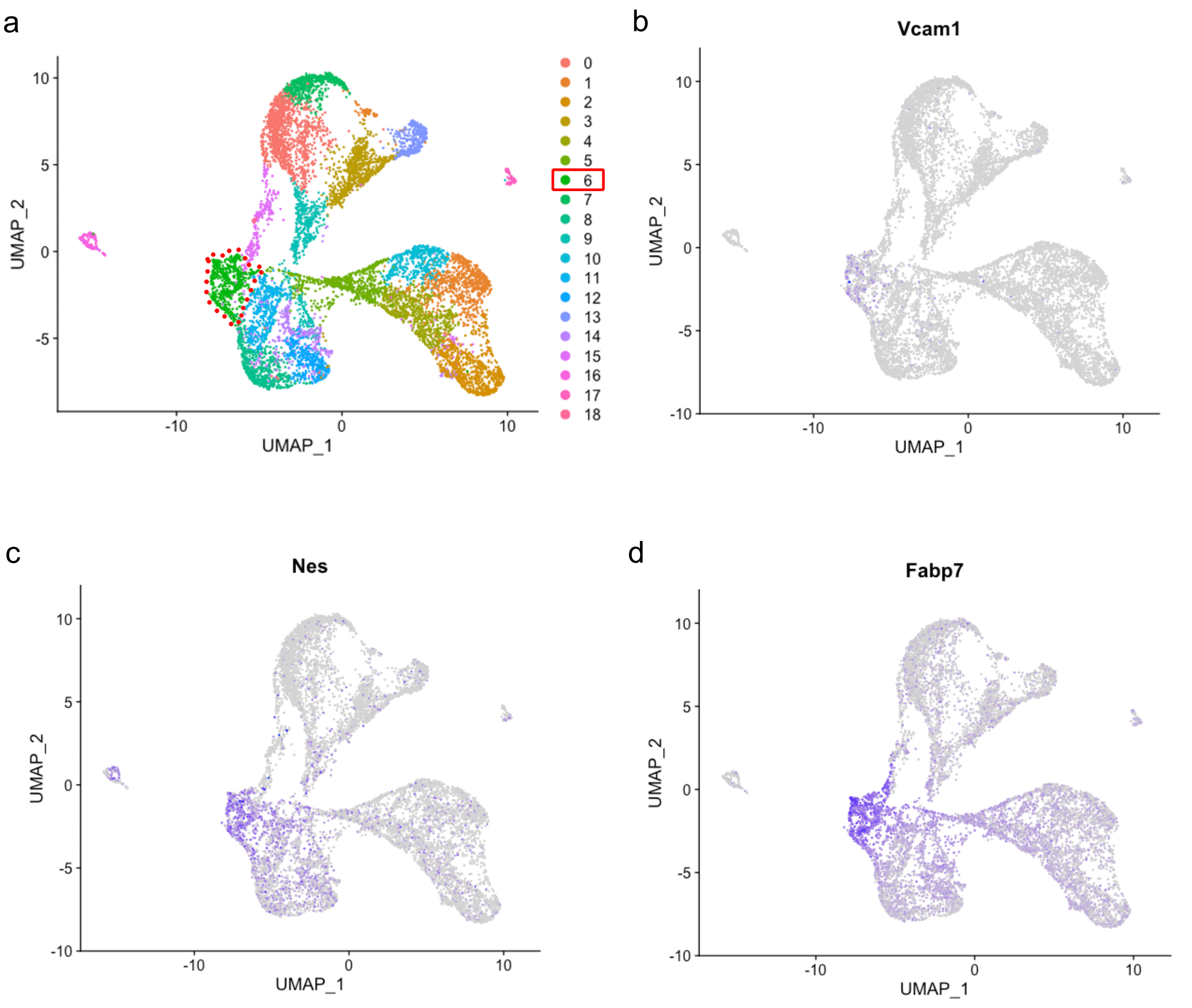

Supplementary Figure 7. Identification of the radial glia cluster in the E14.5 scRNA-seq dataset Cluster 6 (outlined in red) in (**a**) expresses *Vcam1* (**b**), *Nestin* (**c**), and *Fabp7* (**d**) and was identified as the radial glia cluster.

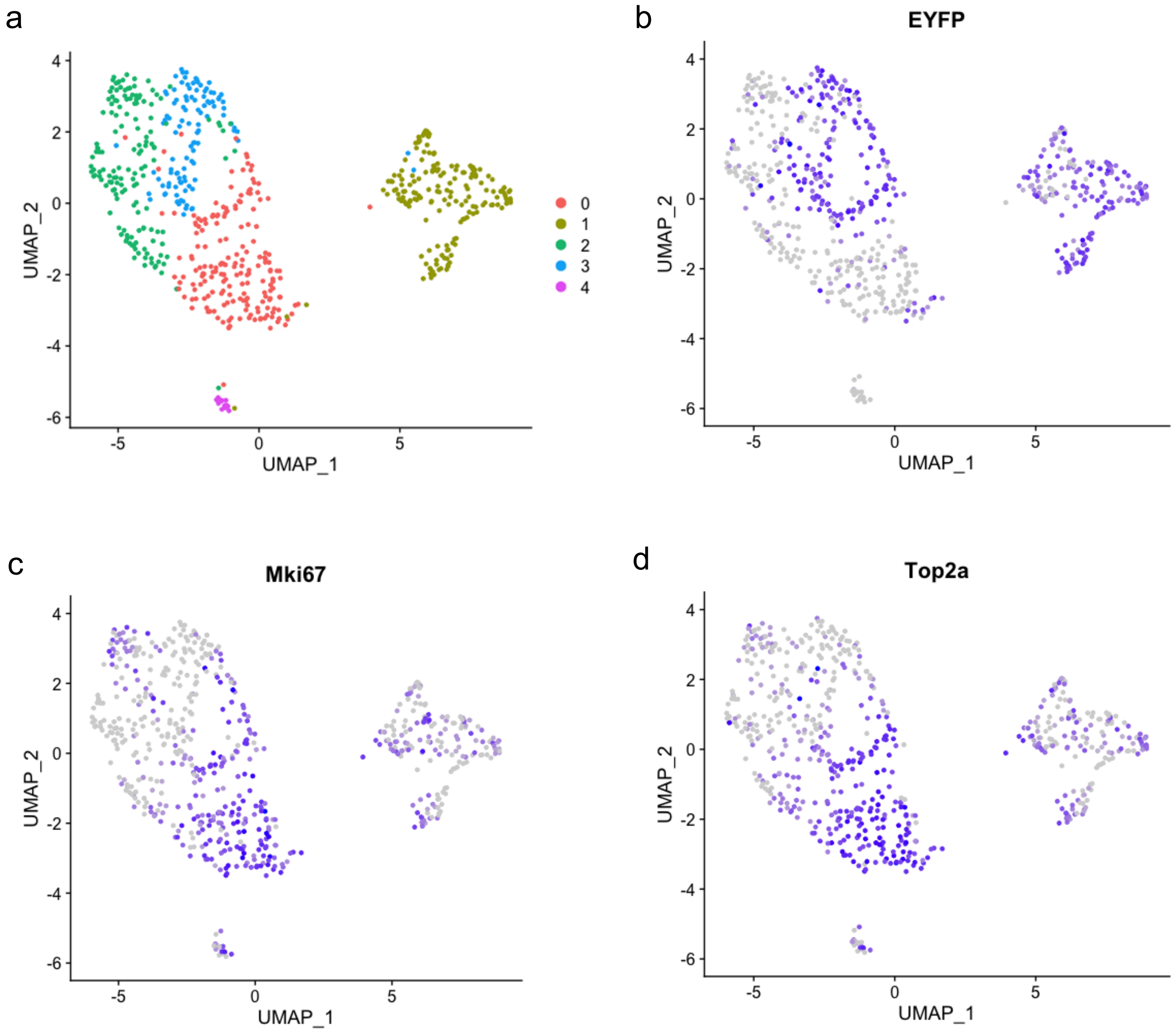

Supplementary Figure 8. Subclusters of radial glia in the E14.5 scRNA-seq dataset
a UMAP showing 5 subclusters of radial glia in the E14.5 scRNA-seq dataset.
b Expression of Emx1-EYFP suggests that subclusters 1 and 3 are dorsal radial glia and subclusters 0 and 2 are lateral radial glia
c, d Expression of the proliferative cell markers Mki67 and Top2a suggests that subcluster 2 are quiescent radial glia and subcluster 0 are activated radial glia.

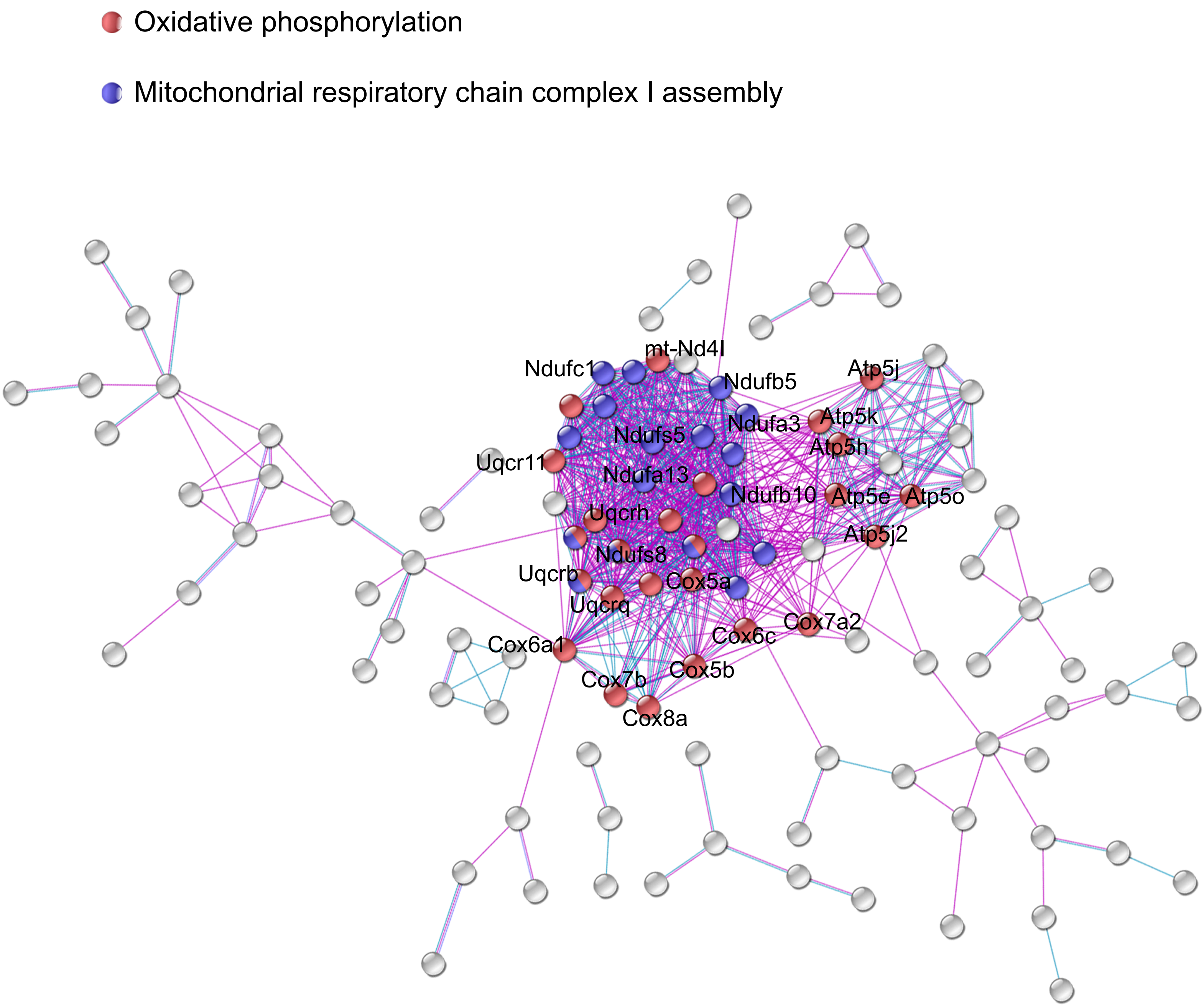

Supplementary Figure 9. Protein-protein interaction network analysis

Protein-Protein interaction networks in the genes upregulated in V-SVZ NSCs from one-month-old *Prdm16* cKO mice. The highlighted GO terms are oxidative phosphorylation (red) and mitochondrial respiratory chain complex I assembly (purple).

Enriched pathways in the upregulated genes

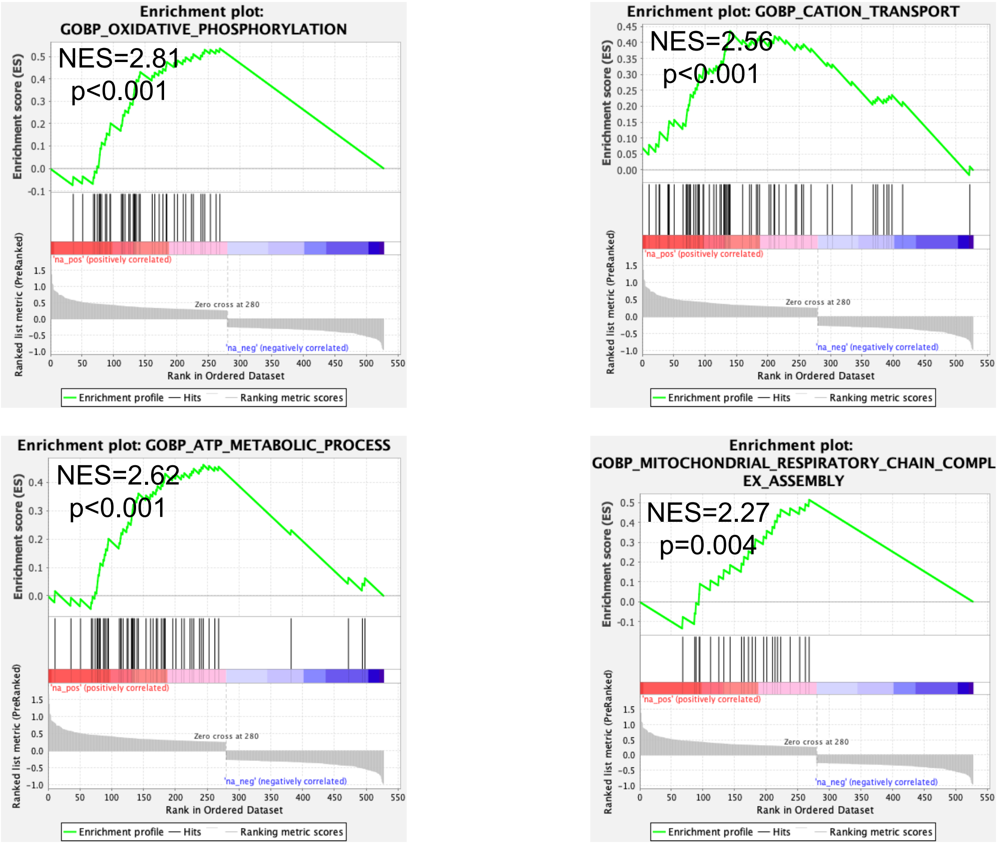

Enriched pathways in the downregulated genes

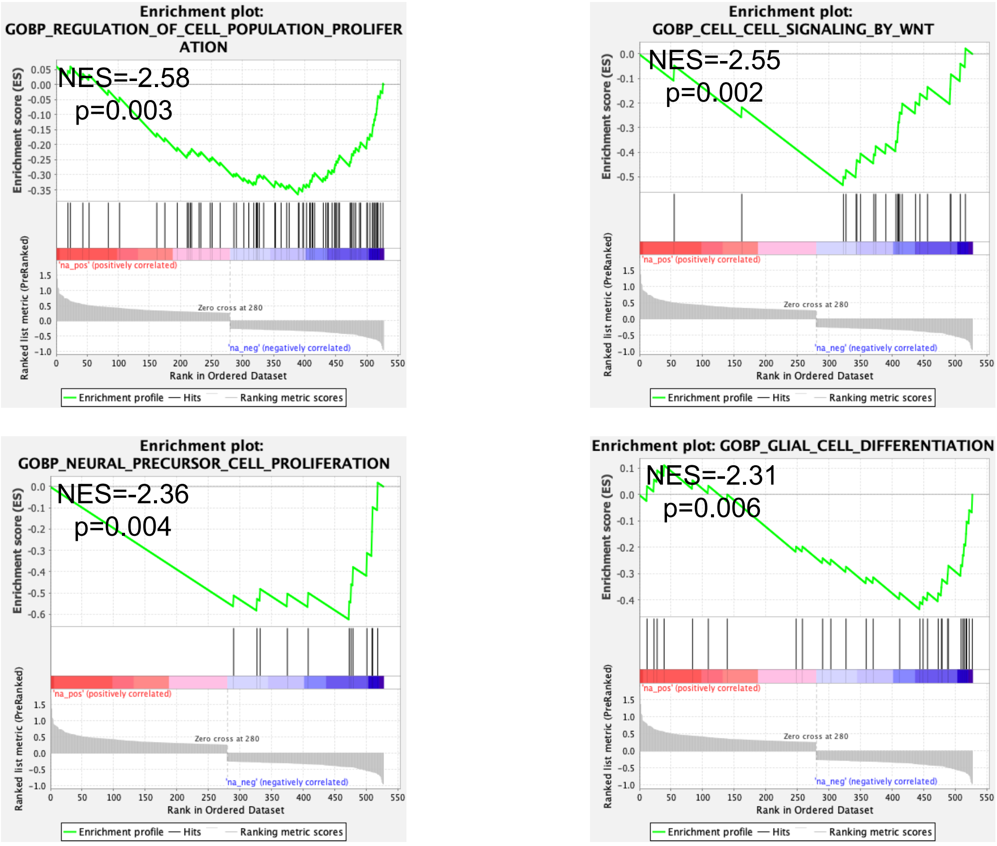

Supplementary Figure 10. Gene set enrichment analysis of differentially expressed genes between one-month-old *Prdm16* cKO and control V-SVZ NSCs

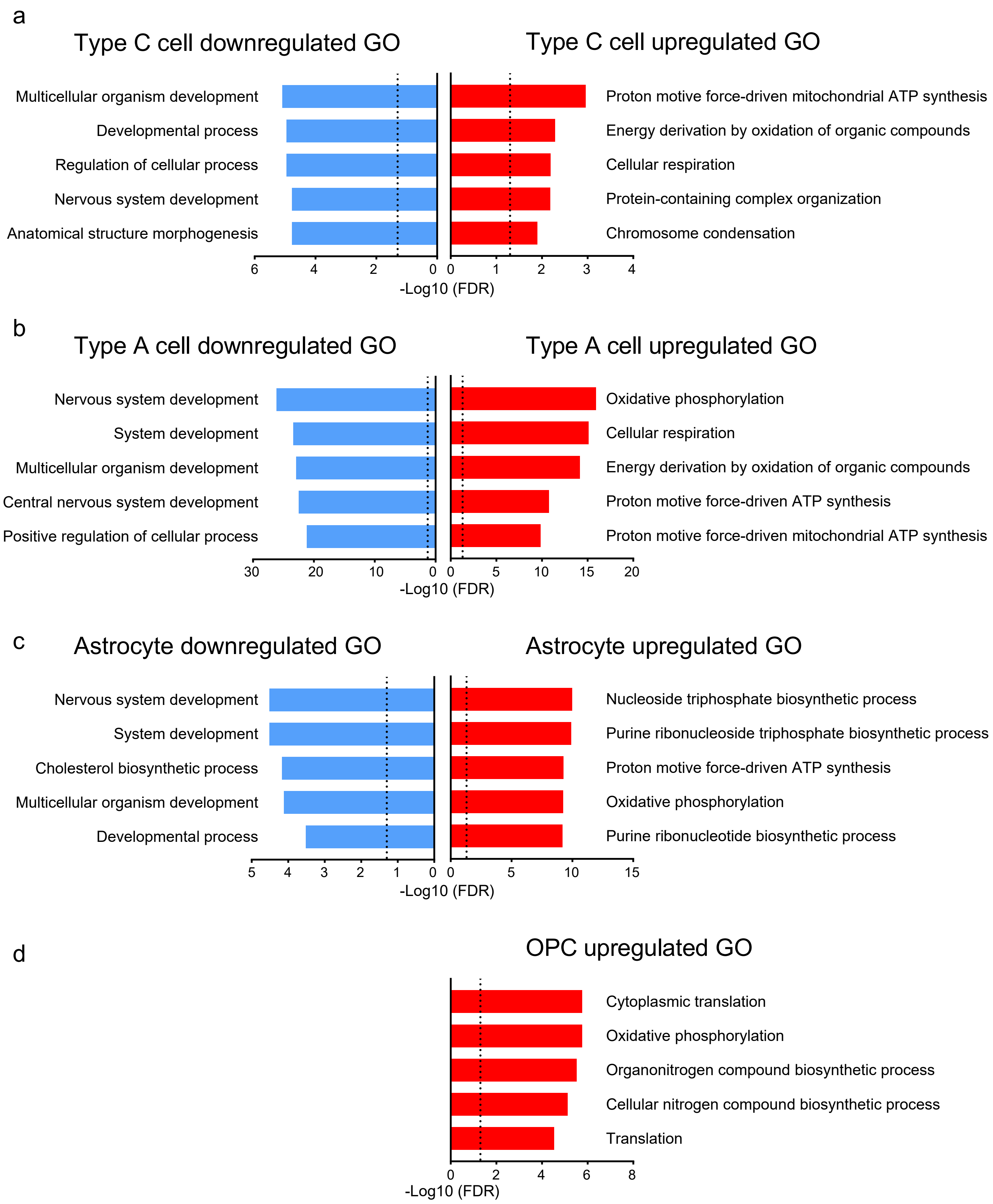

Supplementary Figure 11. GO terms enriched in genes up- and down-regulated in non-NSC cell types in one-month-old *Prdm16* cKO compared to controls.
a type C cells/transiently amplifying precursors (TAPs), **b** type A cells/neuroblasts, **c** astrocytes, **d** oligodendrocyte precursor cells.

a

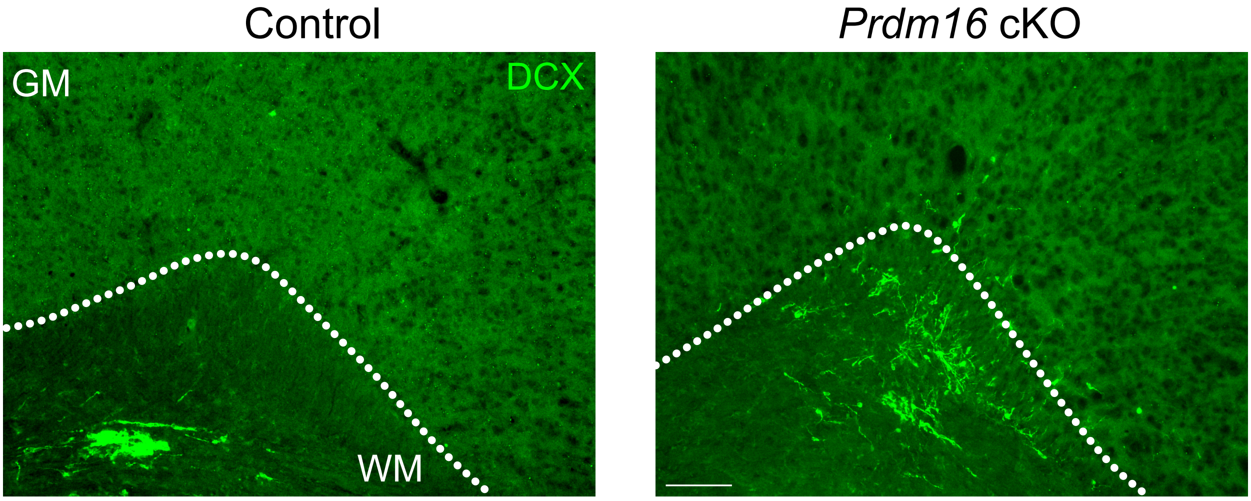

b

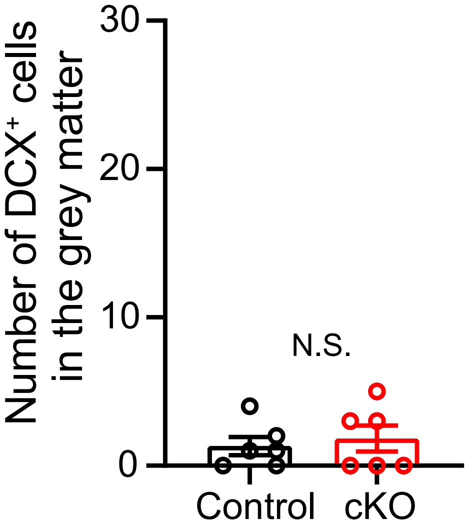

Supplementary Figure 12. DCX⁺ cells in *Prdm16* cKO and control mice at P30.
a Representative images of DCX staining of *Prdm16* cKO and control mice at P30. Dotted lines delineate the boundary between cortical grey matter and white matter (corpus callosum). GM: grey matter. WM: white matter. Scale bar: 100 μm. **b** Quantification of the numbers of DCX^+ cells in the grey matter at P30. n=6 mice per genotype, p=0.6506, two tailed Welch's t-test

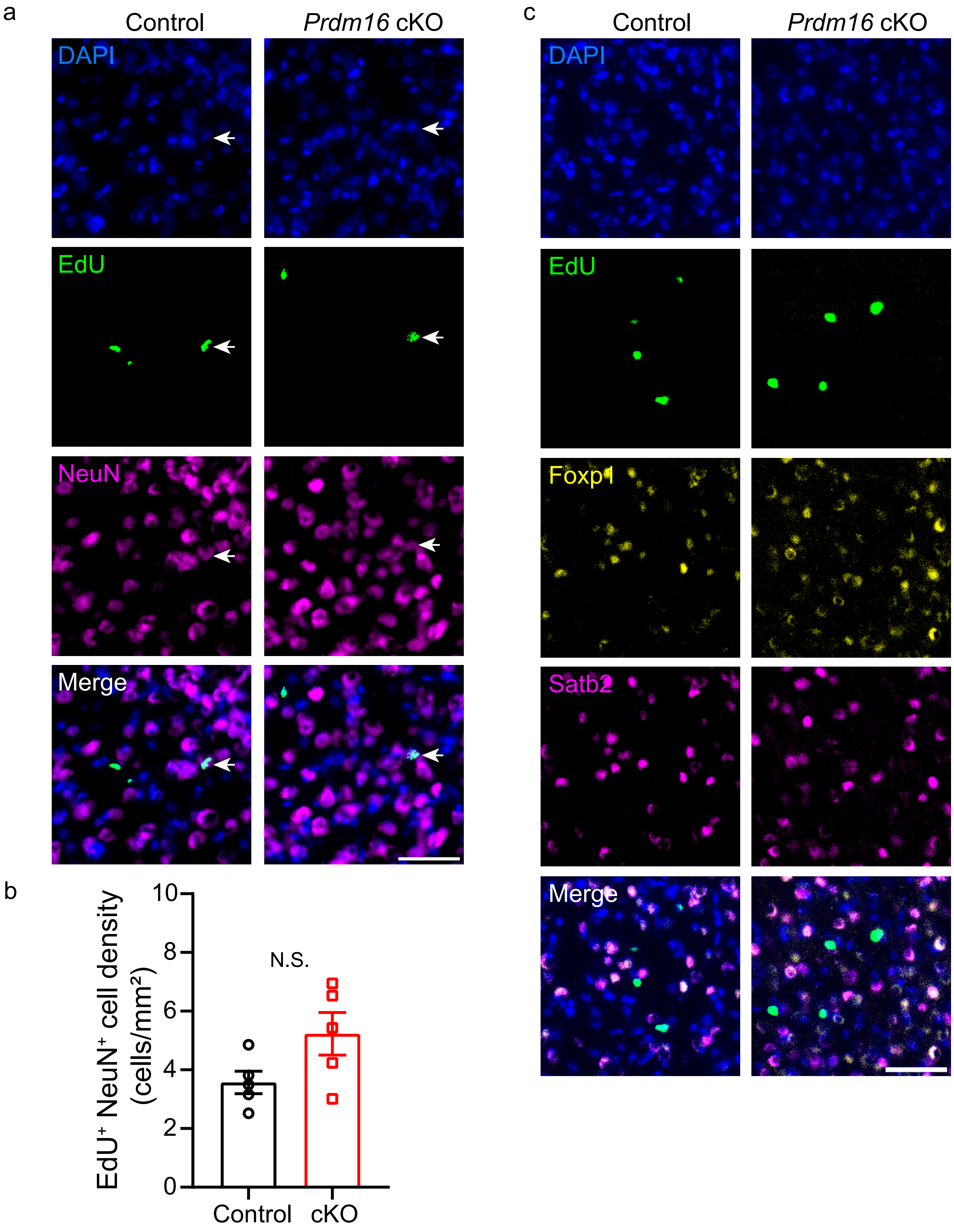

Supplementary Figure 13. Few postnatally born neuroblasts become mature neurons in *Prdm16* cKO mice. EdU was injected at P14 and mice were sacrificed at P28. Mature neuron markers NeuN, Foxp1, and Satb2 were co-labeled with EdU and $EdU^{+}NeuN^{+}$ cell densities in the cortex were quantified. **a** $EdU^{+}NeuN^{+}$ cells were detected at low density in the cortex in *Prdm16* cKO and control mice. Arrows point to examples of $EdU^{+}NeuN^{+}$ cells. Scale bar: 50 μm. **b** Quantification of $EdU^{+}NeuN^{+}$ cell density in the cortex. n=5 mice per genotype, p=0.0894, two tailed Welch's t-test. **c** Co-staining of EdU, Foxp1, and Satb2 in the cortex. Scale bar: 50 μm.

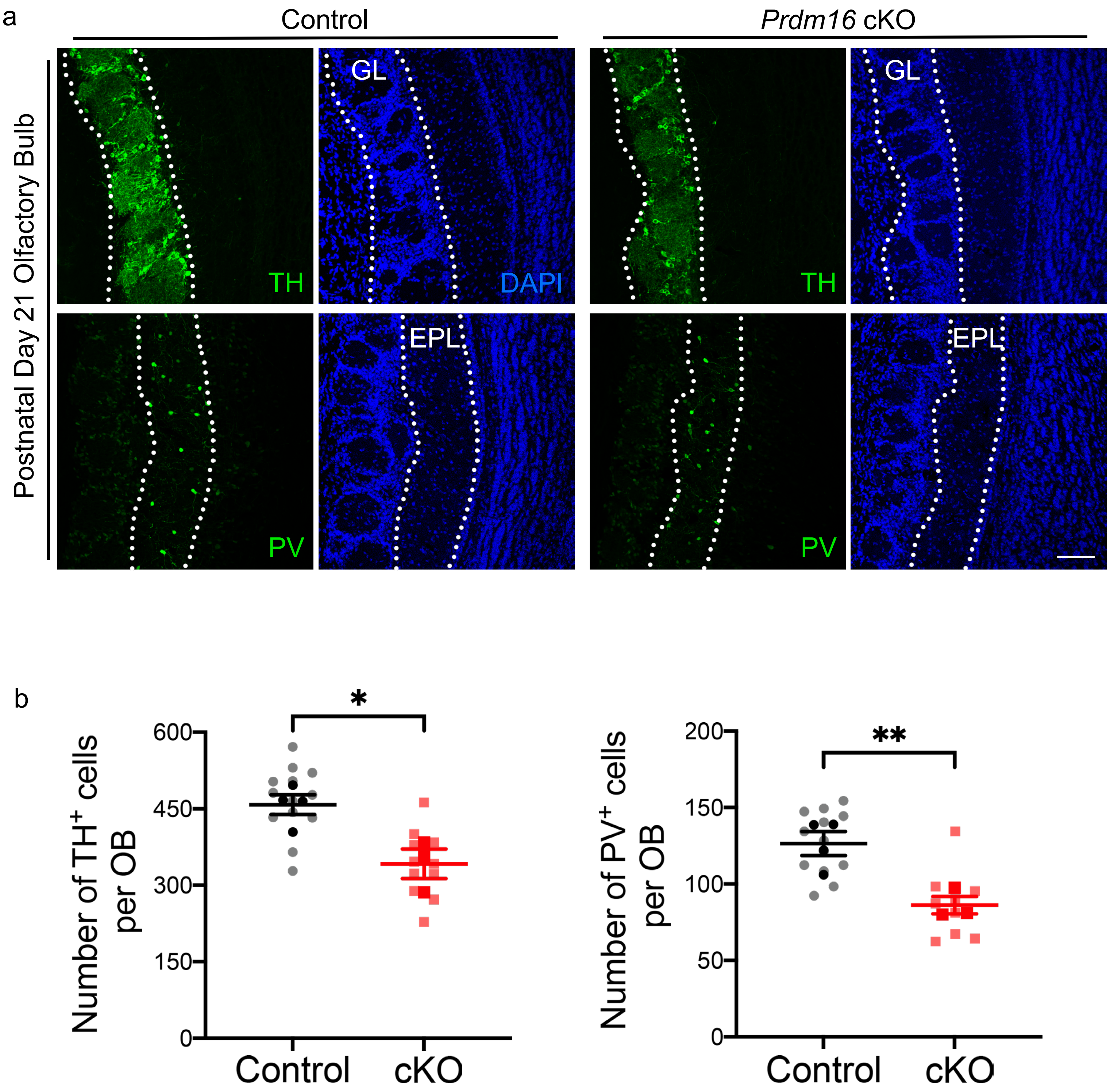

Supplementary Figure 14. Olfactory bulb interneuron development defect in *Prdm16* cKO mice

a Two subtypes of olfactory bulb interneurons. The densities of TH^+ and PV^+ interneurons were reduced in the olfactory bulbs of *Prdm16* cKO mice at P21. The dashed lines delineate the glomerular layer (GL), external plexiform layer (EPL), and granule cell layer (GCL) in the olfactory bulb. Scale bar: 100 µm.

b Quantification of the two subtypes of olfactory bulb interneurons. n=4 mice per genotype. The black and red dots indicate average results from each mouse and the grey and pink dots indicate the raw counts from each image. TH^+ cells: p= 0.0328, two tailed Welch's t-test. PV^+ cells: p=0.0092, two tailed Welch's t-test.

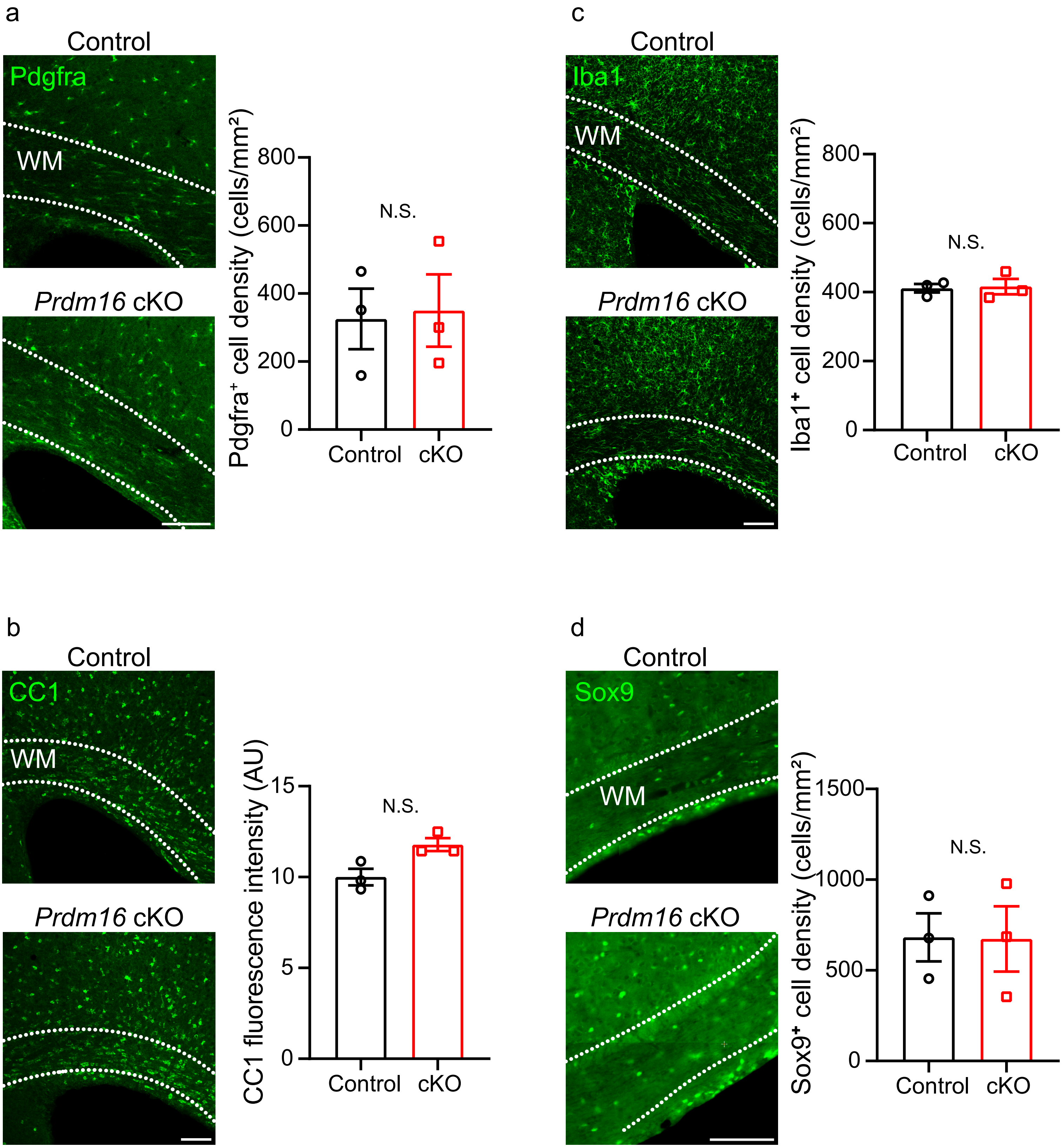

Supplementary Figure 15. No change in the density of glial cells in *Prdm16* cKO mice.
Representative images and quantification of the density of oligodendrocyte precursor cells (Pdgfra$^+$), oligodendrocytes (CC1$^+$), microglia (Iba1$^+$) and Astrocytes (Sox9$^+$) at P21. WM: white matter. Quantification was performed using image of the corpus callosum. All scale bars are 100 µm. Pdgfra: p=0.8683, two tailed Welch's t test. Iba1: p=0.8581, two tailed Welch's t test. CC1: p=0.1000, two tailed Mann Whitney test, Sox9: p=0.9707, two tailed Welch's t test. n=3 mice per genotype.

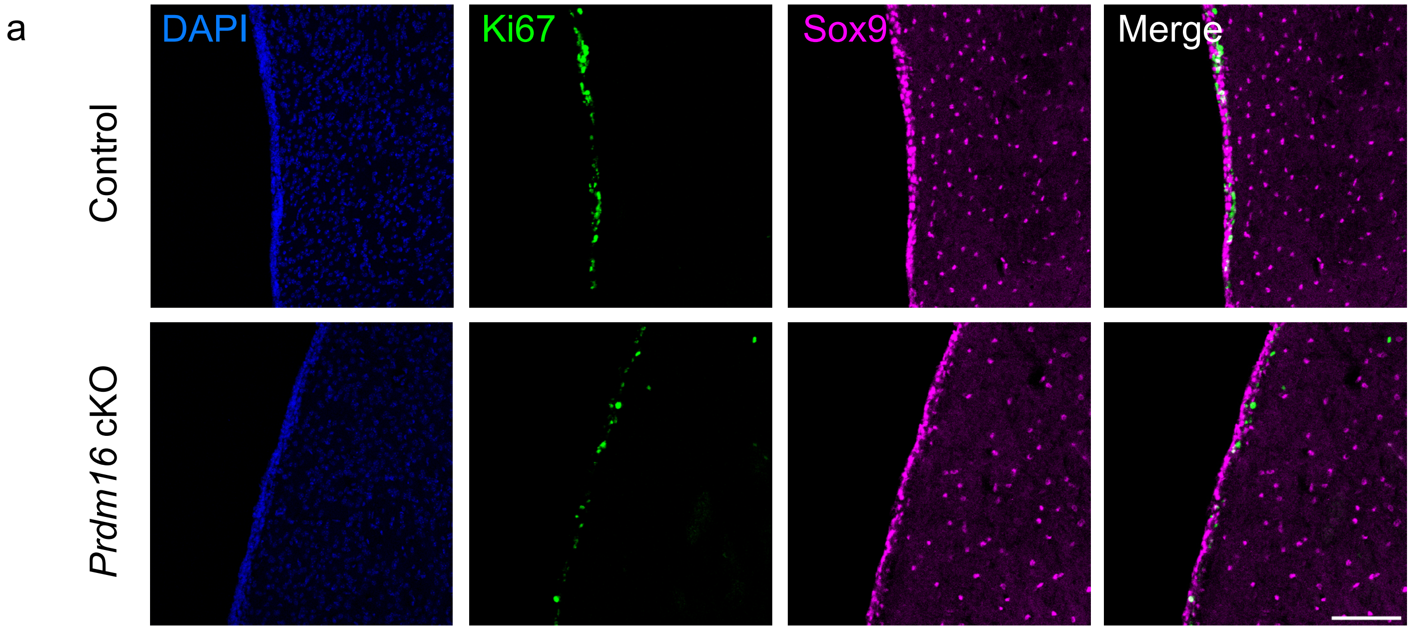

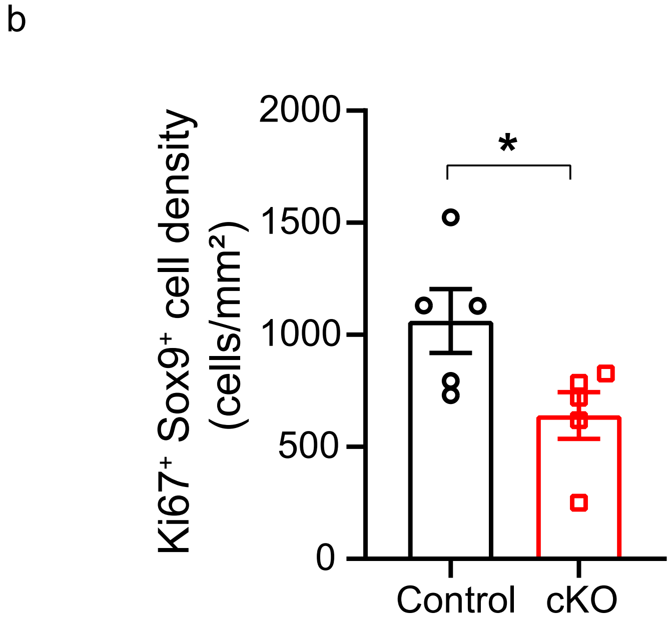

Supplementary Figure 16. A reduction in Ki67$^+$Sox9$^+$ cells in the V-SVZ of P21 *Prdm16* cKO mice.

Representative images (**a**) and quantification (**b**) of the density of Ki67$^+$Sox9$^+$ cells in the V-SVZ of *Prdm16* cKO and control mice at P21. Scale bar: 100 µm. n=5 mice per genotype, p=0.0462, two tailed Welch's t-test.

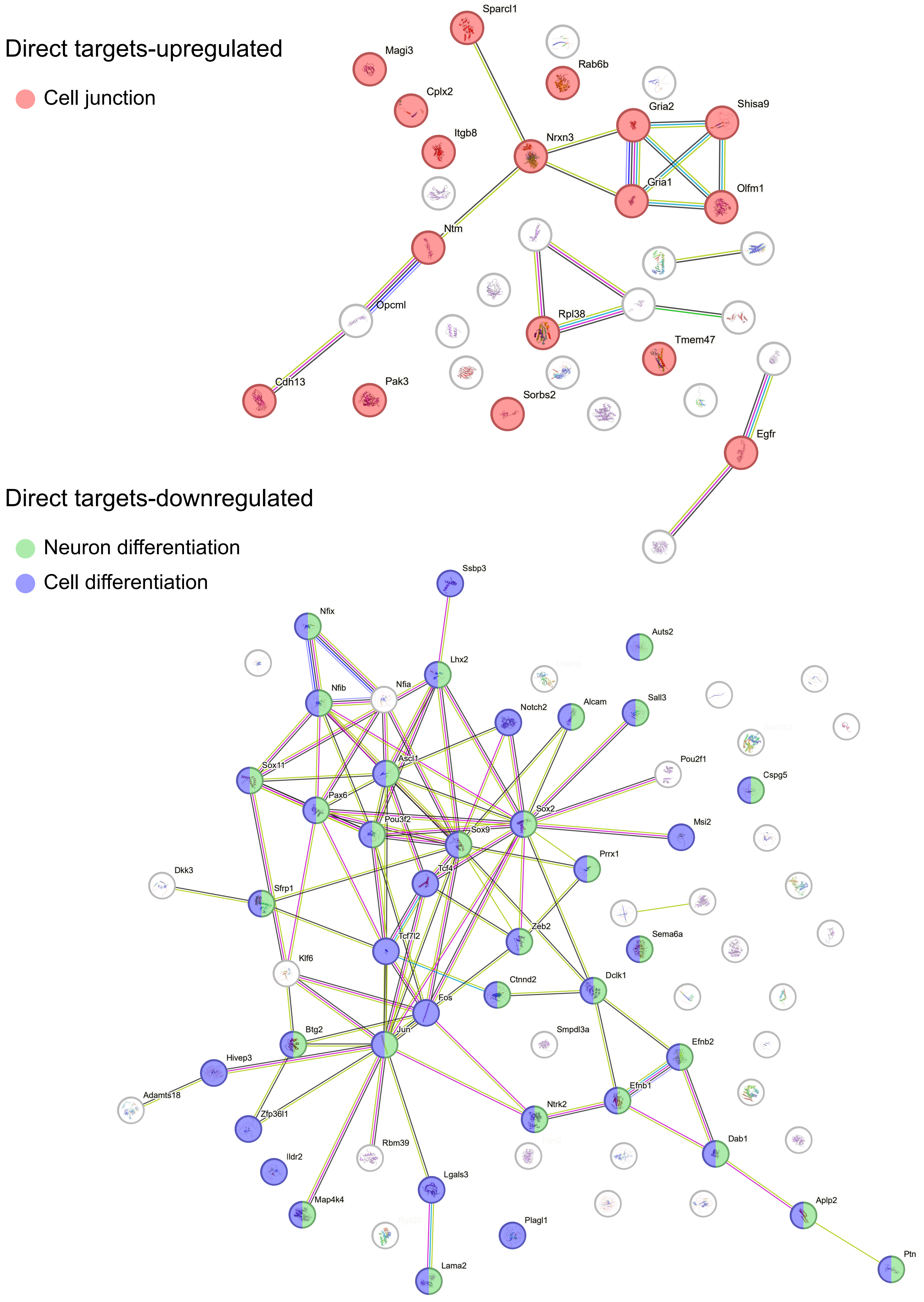

Supplementary Figure 17. Enriched gene ontology terms among direct target genes of Prdm16. Genes that have Prdm16 binding peaks in ChIP-seq and are differentially expressed in scRNA-seq were identified as direct target genes of Prdm16. Gene ontology analysis was performed among up- and down-regulated direct target genes. Protein-protein interaction networks are shown. The top enriched terms were color-coded and highlighted.

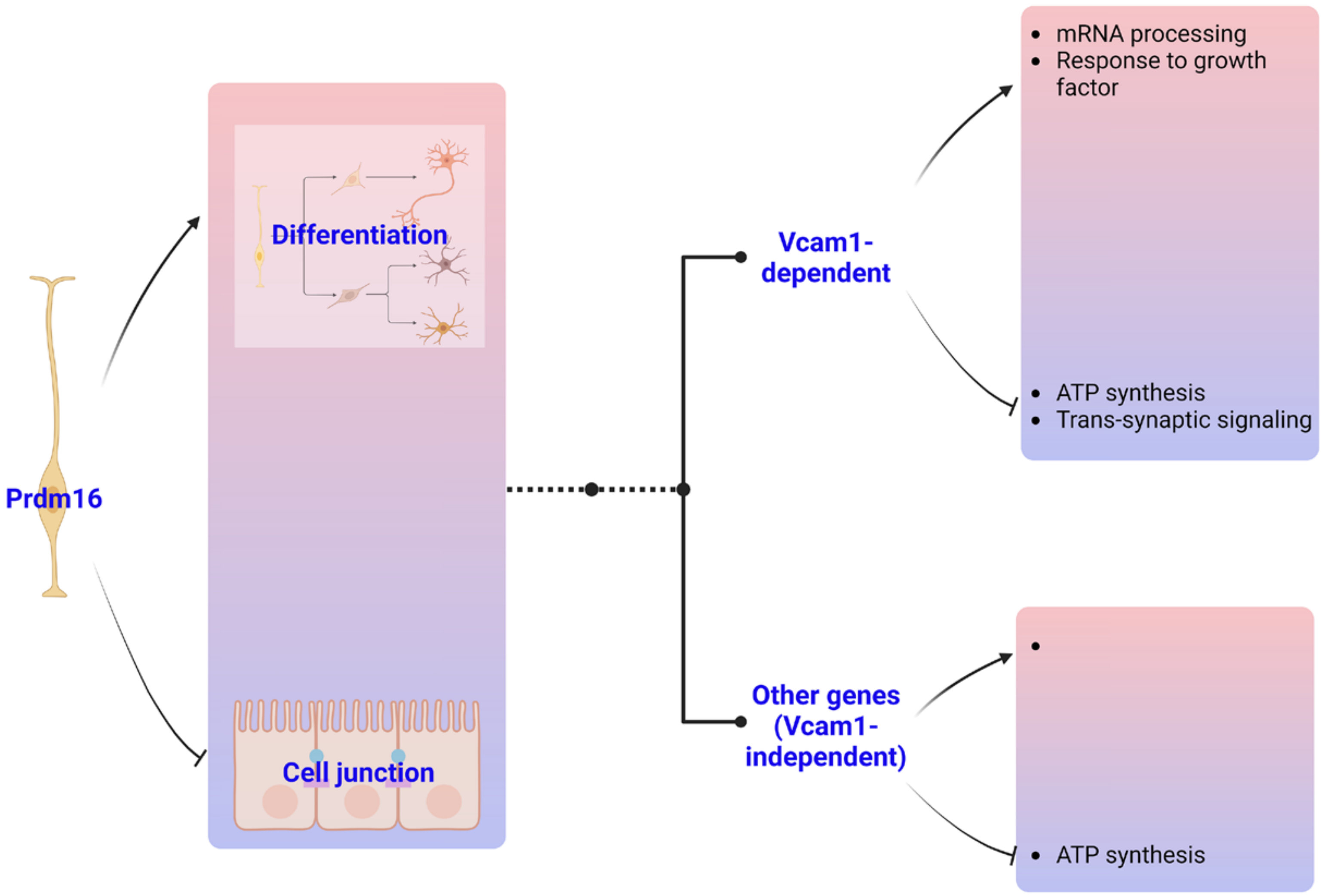

Supplementary Figure 18. A diagram summarizing top enriched gene ontology terms among direct targets of Prdm16 and genes indirectly regulated by Prdm16 in Vcam1-dependent and independent manners.

Created in BioRender. Zhang, Y. (2025) https://BioRender.com/u82e306

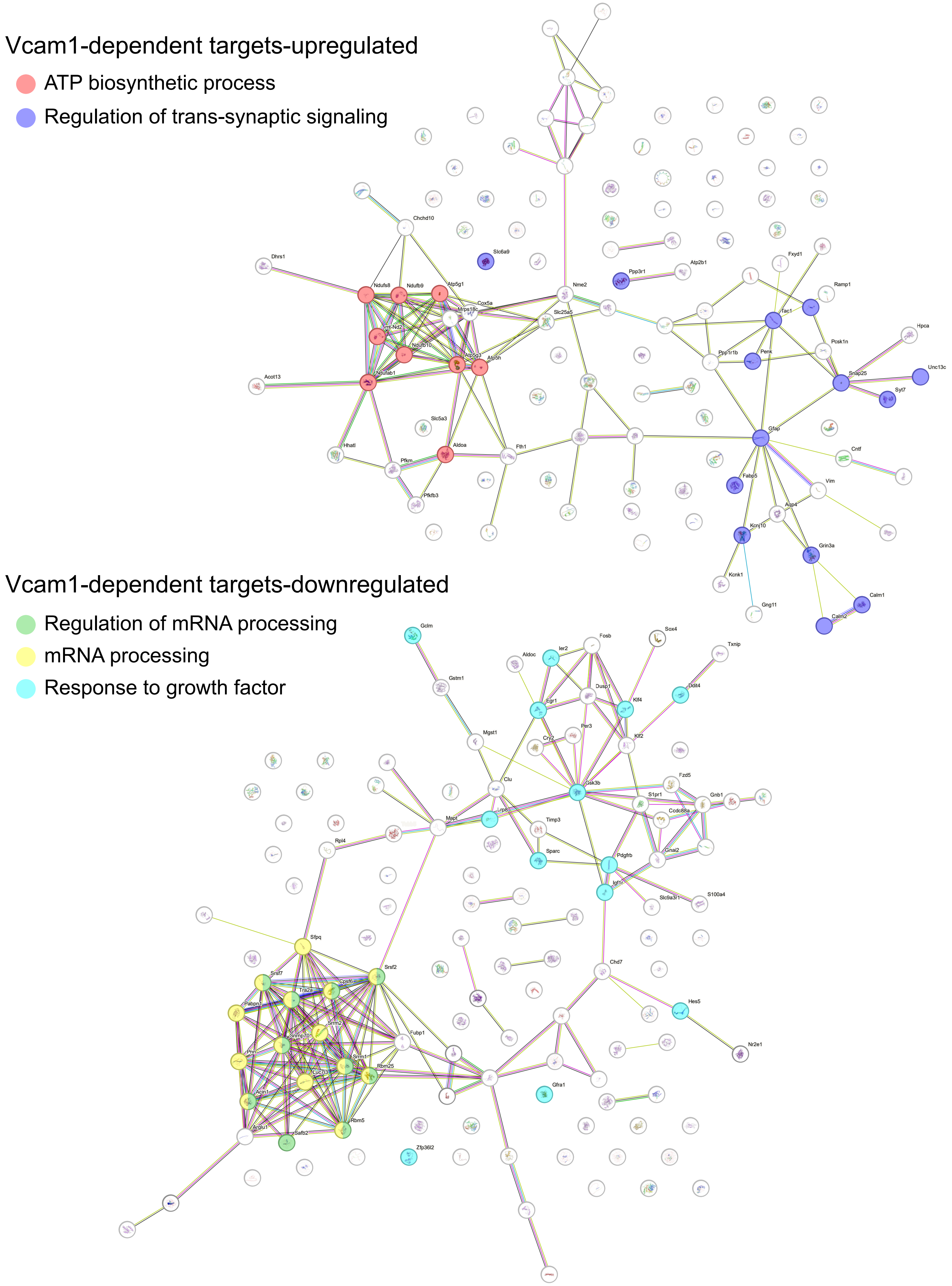

Supplementary Figure 19. Enriched gene ontology terms among genes regulated by Prdm16 in a Vcam1-dependent manner. Genes that are differentially expressed between *Prdm16* cKO and control NSCs and rescued in *Prdm16-Vcam1* DKO NSCs (not significantly different between *Prdm16-Vcam1* DKO and control) were identified as Vcam1-dependent genes. Gene ontology analysis was performed among these Vcam1-dependent genes. Protein-protein interaction networks are shown. The top enriched terms were color-coded and highlighted.

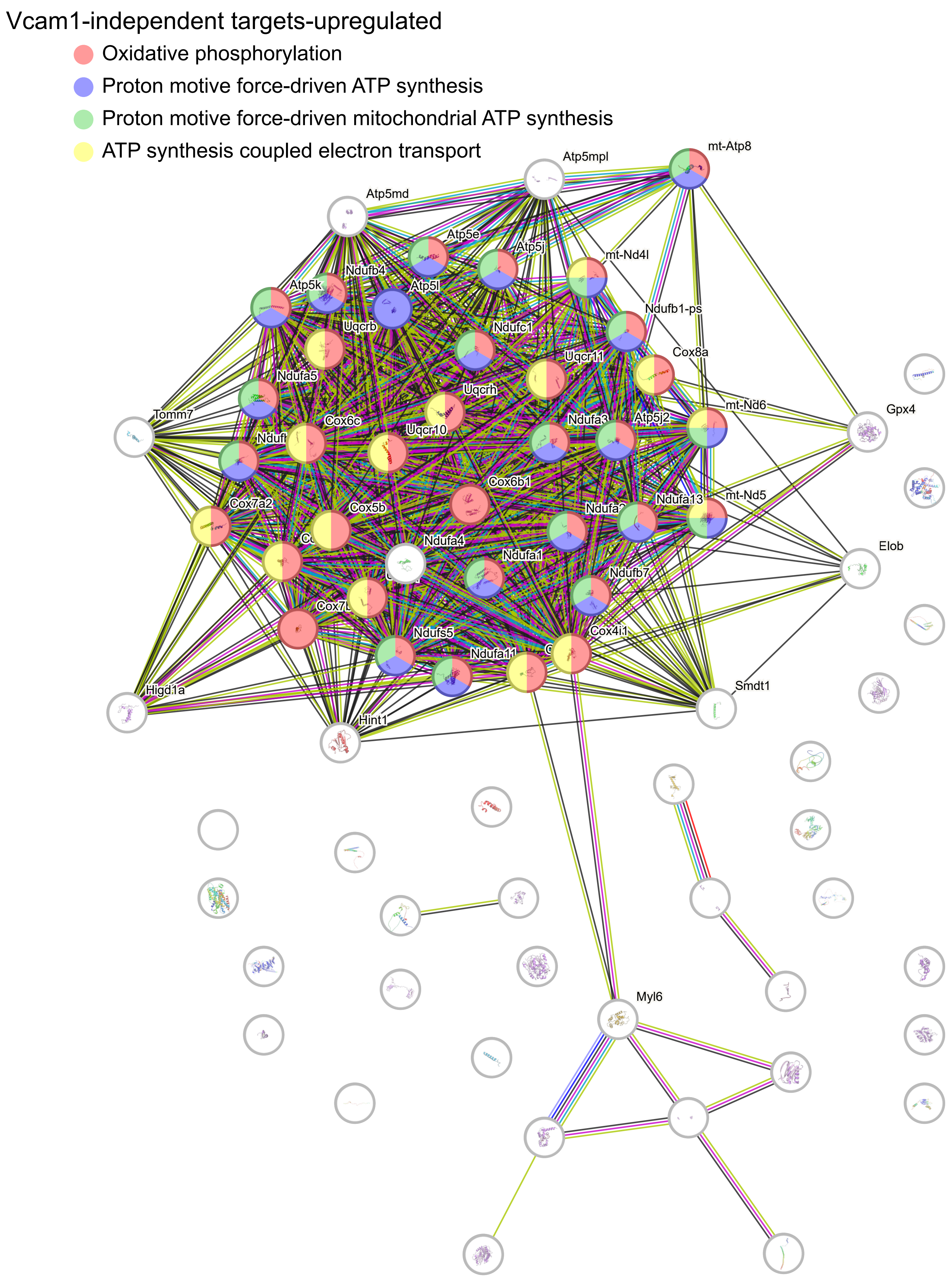

Supplementary Figure 20. Enriched gene ontology terms among genes regulated by Prdm16 in a Vcam1-independent manner. Genes that are differentially expressed between *Prdm16* cKO and control and between *Prdm16-Vcam1* DKO and control in the same direction were identified as Vcam1-independent genes. Gene ontology analysis was performed among these genes. Protein-protein interaction networks are shown. The top enriched terms were color-coded and highlighted.
