# Supplementary material for: Prdm16 regulates the postnatal fate of embryonic radial glia via Vcam1-dependent mechanisms
Source: Nat Commun. 2025 Jul 19;16:6659. doi: 10.1038/s41467-025-60895-y (PMC12276310; doi:10.1038/s41467-025-60895-y)
Supplement: Supplementary file 2 — Description of Additional Supplementary Files [file 41467_2025_60895_MOESM2_ESM.pdf]

Description of Additional Supplementary Files

File name: Supplementary Data 1
Description: Pseudobulk gene expression levels in each ventricular-subventricular zone cell type in one-month-old *PRDM16 cKO*, *PRDM16-VCAM1* DKO, and control mice.
Gene expression is shown as transcripts per million.

File name: Supplementary Data 2
Description: Genes differentially expressed in each cell type in *PRDM16* cKO vs control mice.
P_val, nominal P-value. P_val_adj, P-values adjusted for multiple comparisons. Ave_log2FC, log2 fold change. Pct. 1, percentage of cells expressing the gene in PRDM16 cKO. Pct.2, percentage of cells expressing the gene in control. Wilcoxon test with Bonferroni correction.

File name: Supplementary Data 3
Description: Enriched gene ontology terms in genes up- and down-regulated in NSCs in *PRDM16* cKO compared with control mice.

File name: Supplementary Data 4
Description: Enriched gene ontology terms in genes up- and down-regulated in other cell types in *PRDM16* cKO compared with control mice.
